# Sex Affects the Relationship Between Third Party Punishment and Cooperation

**DOI:** 10.1038/s41598-019-40909-8

**Published:** 2019-03-12

**Authors:** Claudia Rodriguez-Ruiz, José Antonio Muñoz-Reyes, Marta Iglesias-Julios, Santiago Sanchez-Pages, Enrique Turiegano

**Affiliations:** 1 Biology Department, Universidad Autónoma de Madrid. C/Darwin 2, 28029 Madrid, Spain; 2 Laboratorio de Comportamiento Animal y Humano, Centro de Estudios Avanzados, Universidad de Playa Ancha, Valparaíso, Chile; 3 Champalimaud Neuroscience Programme, Champalimaud Centre for the Unknown, Lisbon, Portugal; 4 Department of Political Economy, King’s College London, London, UK

## Abstract

Prosocial third-party punishment (3PP) is a punitive behavior against antisocial individuals, which might explain extended cooperativeness in humans. 3PP shows sexual dimorphism, being more frequent in men than in women. We studied whether sexually dimorphic features related to sexual hormones during development (facial dimorphism and 2D:4D) influence the tendency to engage in 3PP in a sample of 511 women and 328 men. After playing a Prisoner’s Dilemma, participants had to decide whether to penalize the defection of a third player who had exploited his/her counterpart’s cooperation. In line with previous studies, we observe that men are more prone to engage in 3PP than women. We find that this sex difference is due to cooperative men being more likely to punish than cooperative women. In addition, men with higher facial masculinity are less likely to engage in 3PP, whereas no features influence 3PP in women. We discuss the possibility that sex differences in the motivations and fitness implications underlying 3PP might be driving the observed results.

## Introduction

Third party punishment (3PP) is a behavior costly to the individual exerting it and aimed at penalizing individuals who have not directly interacted with the punisher. It is considered to be altruistic when individuals exert it to penalize others’ harmful behavior^1^. Altruistic 3PP appears in many human societies^2–4^. Because of that, it has been proposed as one among the possible explanations for extended human cooperation^1,5,6^, as individuals who engage in altruistic 3PP pay currently a cost to increase social welfare. Still, for altruistic 3PP to be selected for, it must improve the inclusive fitness of individuals exerting it^7^; either directly (i.e. increasing reproductive success through processes linked to reciprocity or reputation) or indirectly^8–11^. In fact, the attainment of such fitness benefits must be the ultimate cause of prosocial behavior^7,12,13^, independently of whether the individual receives in exchange future social advantages or an improvement in resources. From this viewpoint, altruistic 3PP is expected to share proximal and evolutionary motivations and influences with other forms of prosociality. Individuals who engage in altruistic 3PP should behave prosocially in different contexts^4^, assuming that the tendency to behave prosocially carries over different situations^2,14–18^, and across time^14,19,20^.

Developmental and physiological features have been found to influence cooperation^21–25^, trustworthiness^26^ and spontaneous generosity^27^. Thus, it is likely that 3PP is also influenced by these features, which seem to link the genetic propensity to behave prosocially, established throughout the evolutionary process^7,28^, to actual prosociality. Genes, jointly with the developmental environment, affect various biological features that in turn influence behavior. These features include hormonal levels, specific neural properties or preconfigured neural circuits, and even physiological features not directly related to behavior but with an indirect impact on it. Sex has been the only biological variable previously studied in relation to 3PP: it has been shown that men and women engage in 3PP with different intensity, with men punishing more harshly than women^15,29,30^. On the other hand, meta-analyses show that there is no such sexual dimorphism in cooperative behavior^31,32^, indicating that the relationship between cooperation and 3PP might be more complex than previously thought.

Among the physiology-related variables with a likely influence on 3PP, those related to cooperation are good candidates^21–25^ given the described association between altruistic 3PP and the tendency to cooperate^33–35^. Interestingly, some of these variables are sexually dimorphic^36,37^, or show differential impacts on cooperation by sex^25,38,39^. Therefore, these features might be behind the described sexual differences in 3PP. Our aim in this work is to study for the first time how features related to individual development influence the tendency to engage in altruistic 3PP in the context of a social dilemma. In addition, we study whether these features can also explain the observed sex differences in altruistic 3PP.

Cooperative behavior is affected by the levels of sexual hormones to which an individual is exposed during development^21,22,26,40–42^. Their influence is due to their organizational effects during this period, in which they conform and modify the anatomy and physiology of growing organisms^43,44^, including the nervous system^45^. They can affect adult behavior through this influence on the development of the nervous system. Sexual hormones have crucial organizational effects during two periods: prenatal and pubertal^44,45^. The exposure to sexual hormones during these periods can be proxied with two widely-used morphological features: the second-to-fourth digit ratio (2D:4D) and facial masculinity/femininity^46,47^.

2D:4D is influenced by the prenatal ratio of testosterone/estrogen activity^36^. Lower ratios are indicative of higher exposure to testosterone during human prenatal development^48^. Although this relationship has become controversial in recent years^49,50^, there is still solid evidence of a link between prenatal hormone levels and 2D:4D^36,51,52^. The association between 2D:4D and prosocial behaviors (e.g. generosity, rejection of injustice) has been described in diverse experimental settings^21,27^. However, the link between 2D:4D and cooperative behavior is not fully understood yet. High values of 2D:4D are related to less cooperation in both sexes. Results are mixed though for high levels of cooperation, which have been associated to both medium and low 2D:4D values^21,22^. These conflicting results might be accounted for by the role of 2D:4D as a modulator of the effect of other variables on behavior^27,53,54^.

Facial dimorphism, usually referred to as facial masculinity/femininity in the literature, is a proxy for pubertal exposure to sexual hormones^47^, with higher values of dimorphism indicating higher levels of estrogens and testosterone levels for women and men, respectively. Higher facial masculinity is negatively related to prosociality in men^26,40–42^ (depending on the social context^23^), as it is associated in the laboratory with aggression^41^, unethical behavior^26^, and cheating in negotiations^42^. No correlation has been found between facial femininity and prosociality in women^25^.

Another developmental variable with an impact on cooperation is facial fluctuating asymmetry (FA). This measure is related to developmental instability^55^, and it is frequently employed as a proxy for phenotypic quality^56^, although with some limitations^57^. More asymmetrical individuals are more cooperative and engage more often in prosocial behaviors^22,25,39^, albeit these effects are stronger in men. FA is closely related to attractiveness^58,59^, which in turn has been shown to be negatively related to prosociality in women^25,60,61^ but positively in men^38^.

In this work, we explore whether physiology related variables influence altruistic 3PP in the context of a social dilemma, the Prisoner’s Dilemma Game (PDG), and whether these variables can explain the sex differences observed in this form of punishment. We expect cooperative individuals, both men and women, to be more likely to engage in altruistic 3PP (*Hypothesis 1*). On the basis that altruistic 3PP is related to cooperation, we entertain three additional hypotheses. We expect higher values of 2D:4D to correlate with a lower tendency to engage in 3PP (*Hypothesis 2*), given that cooperativeness is in turn negatively related with 2D:4D. We also expect men with lower facial dimorphism to engage in 3PP more often, but not women (*Hypothesis 3*), as facial dimorphism has a differential effect on cooperation by sex. Finally, we expect more asymmetrical men and women to exert 3PP more often given that FA is positively correlated with cooperation, although this association should be stronger in men (*Hypothesis 4*). In addition, we test differences in the aforementioned variables between participants who cooperated and those who did not. The aim of our study is to broaden our knowledge of the variables that influence prosociality and the persistence of social norms in order to better understand the causes behind the differences across human social organization systems.

## Results

### Sexual differences in cooperation and 3PP

67.32% of women and 62.20% of men in our sample chose to cooperate; sex differences were not significant (Χ^2^_1_ = 2.32; p = 0.128; φ = 0.05). Regarding expected behavior, 68.49% of women and 65.24% of men believed that their counterpart would cooperate. Sex differences in these expectations were not significant either (Χ^2^_1_ = 0.96; p = 0.328; φ = 0.03). Cooperative behavior correlated strongly with expected cooperation of the counterpart (women: Χ^2^_1_ = 135.73; p < 0.001; φ = 0.52; men: Χ^2^_1_ = 69.66; p < 0.001; φ = 0.46).

As observers, 20.35% of women decided to punish the defector who expected her counterpart to cooperate. This percentage was 26.22% in men. Sex differences in 3PP frequency were significant (Χ^2^_1_ = 3.93; p = 0.048; φ = 0.14).

### Relationship between cooperation, beliefs about the other’s behavior and 3PP

There was no relationship between cooperation and 3PP, either in men (Χ^2^_1_ = 2.04; p = 0.154; φ = 0.08), or women, although the latter was close to significance (Χ^2^_1_ = 3.52; p = 0.061; φ = 0.09). We found no relationship either between the counterpart’s expected behavior and 3PP (women: Χ^2^_1_ = 2.93; p = 0.087; φ = 0.08; men: Χ^2^_1_ = 0.67; p = 0.412; φ = 0.05). However, 28.92% of male cooperators engaged in 3PP whereas only 18.02% of female cooperators did so (Χ^2^_1_ = 8.84; p = 0.003; φ = 0.12). There were no sex differences in the proportion of defecting participants who engaged in 3PP (Χ^2^_1_ = 0.45; p = 0.503; φ = 0.04). Values of these different proportions are included in Supplementary Table S1.

**Figure 1:**
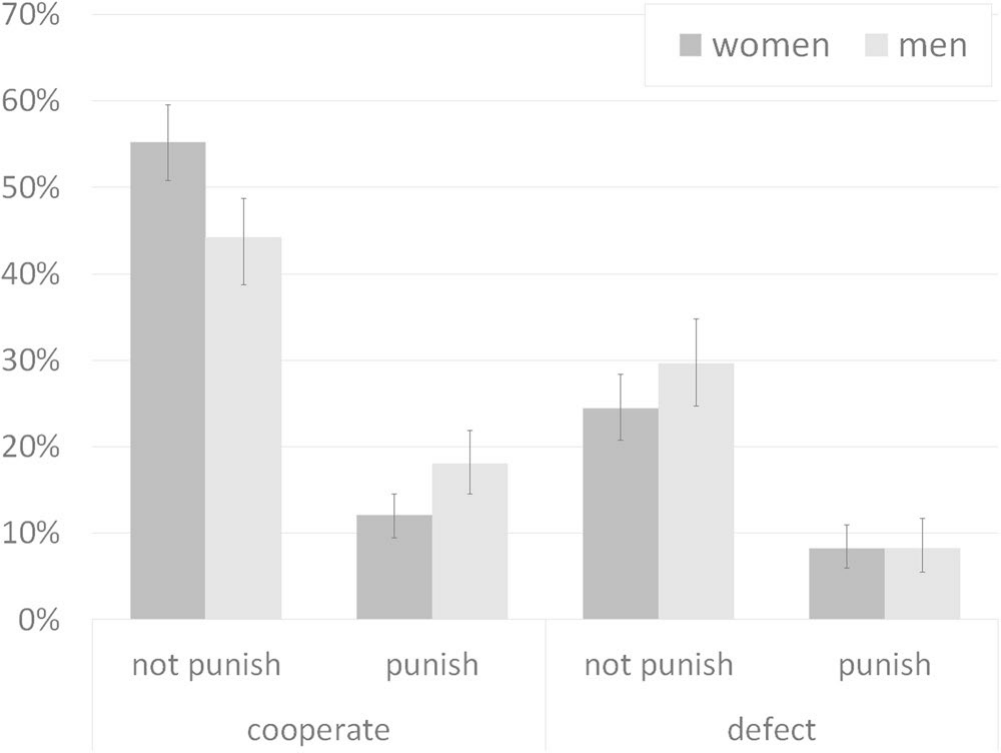
Proportion of male and female participants according to their 3PP and PDG choices. The bars represent the 95% confidence interval for the proportion.

We classified participants in four categories according to their behavior in the two stages, PDG and 3PP (Fig. 1). There were sex differences in the distribution of subjects across these four categories (Χ^2^_3_ = 11.45; p = 0.010; w = 0.12).

### Influence of biological variables on the tendency to punish third parties

We next analysed the influence of biological variables on 3PP by sex. Firstly, we checked for differences in men according to their punitive behavior. We found significant differences (Table 1) in facial dimorphism and self-perceived attractiveness (SPA). In women, we found no significant differences in any physiological variable between those who punished and those who did not.

**Table 1:** Mean values of variables according to participants’ behavior in 3PP and PDG by sex. Data are presented as: mean (95% confidence interval).

	No Punish (N = 407)	Punish (N = 104)	Defect (N = 167)	Cooperate (N = 344)
WOMEN				
Dimorphism	4.54 (4.18–4.75)	4.78 (4.32–5.64)	4.70 (4.23–5.26)	4.54 (4.18–4.79)
	U = 19010, p = 0.109, d = −0.079	U = 27312, p = 0.367, d = 0.053		
FA	3.04 (2.94–3.03)	3.04 (2.91–3.11)	3.02 (2.91–3.04)	3.05 (2.94–3.06)
	U = 20905, p = 0.848, d = 0.001	U = 28379, p = 0.826, d = −0.048		
2D:4D	0.977 (0.973–0.979)	0.982 (0.975–0.989)	0.978 (0.973–0.981)	0.977 (0.974–0.981)
	U = 19158, p = 0.136, d = −0.182	U = 28469.5, p = 0.871, d = −0.021		
SPA	0.612 (0.589–0.639)	0.591 (0.570–0.616)	0.628 (0.610–0.647)	0.599 (0.576–0.619)
	U = 23260, p = 0.119, d = 0.148	U = 25168, p = 0.023, d = 0.203		
	**No Punish (N** = **242)**	**Punish (N** = **86)**	**Defect (N** = **124)**	**Cooperate (N** = **204)**
**MEN**				
Dimorphism	4.96 (4.57–5.32)	4.06 (3.30–4.85)	4.84 (4.29–5.43)	4.66 (4.23–5.08)
	U = 12019, p = 0.033, d = 0.294	U = 12182, p = 0.576, d = 0.059		
FA	2.86 (2.79–2.89)	2.77 (2.67–2.84)	2.82 (2.74–2.88)	2.85 (2.76–2.88)
	U = 11675, p = 0.093, d = 0.204	U = 10659, p = 0.017, d = −0.067		
2D:4D	0.965 (0.961–0.969)	0.965 (0.959–0.972)	0.967 (0.962–0.973)	0.964 (0.960–0.968)
	U = 10318, p = 0.907, d = −0.011	U = 11741, p = 0.276, d = 0.127		
SPA	0.592 (0.570–0.633)	0.657 (0.638–0.711)	0.587 (0.561–0.636)	0.623 (0.593–0.648)
	U = 8204, p = 0.004, d = −0.339	U = 10900.5, p = 0.036, d = −0.186		

We then pooled all observations and built a logistic model (the complete procedure followed is described in Supplementary Tables S2–S4). The final model included the variables sex, behavior in the PDG (PDGb), facial dimorphism and SPA, and their interactions with the sex dummy (Table 2). All variables showed a significant effect on punishing behavior mediated by sex. According to the estimates from the model, a man who cooperates is in average 1.27 times more likely to engage in 3PP compared with a defector, while a woman cooperator is in average 0.70 times more likely to punish than a woman who defected. On the other hand, the estimated risk ratios yield that men with a SPA one standard deviation over the mean are 1.26 times more likely to engage in 3PP, while women in the same situation are 0.85 times more likely to do so. In addition, men with one SD over the mean dimorphism are 0.76 times more likely to punish, while having one SD over the mean has a very weak effect on the likelihood of women engaging in 3PP (they are 1.06 times more likely).

**Table 2:** Final logistic model for 3PP. The variables initially included were Dimorphism (facial dimorphism), SPA (self-perceived attractiveness), and the categorical variables sex and PDGb (behavior in the PDG). The reference values for the categorical variables are male for sex and cooperate for PDGb.

Variables in the model	MODEL				VARIABLE				
	−2LL	Likelihood Ratio Test	Df	p	Variables	Coef	Wald	Df	p
*Sex, PDG b, Dimorphism, SPA, Sex x PDG b, Sex x Dimorphism, Sex x SPA*	871.83	11.88	3	0.007	Constant	−1.662	11.33	1	0.001
					Sex	0.742	1.15	1	0.284
					PDG b	−0.312	1.31	1	0.252
					Dimorphism	−0.109	6.52	1	0.011
					SPA	1.969	7.80	1	0.005
					Sex x PDG b	0.769	4.67	1	0.031
					Sex x Dimorphism	0.139	6.17	1	0.013
					Sex x SPA	−3.208	9.40	1	0.002

### Additional analysis: relationship between biological variables and cooperation

Given the differences in the relationship between cooperation and 3PP in men and women, we also checked for sex differences for cooperators and defectors separately in each of the variables considered (Table 1). There are no significant differences in expected behavior (Supplementary Table S5).

## Discussion

One important result emerging from our analysis is that cooperation and 3PP are not associated equally in men and women. Although it is well described that men are harsher third-party punishers^29^, no previous study has analysed the relationship between 3PP and sex controlling for cooperative behavior. A lack of correlation between cooperation and 3PP was previously described^14^, but without controlling for sex. In contrast, we find that cooperative men are more likely to punish defectors (risk ratio: 1.27), but cooperative women are not (risk ratio: 0.70). We observe this even after controlling for dimorphic variables which could explain this sex difference. In short, our *Hypothesis 1* is only supported for men. It is noteworthy that our design was aimed to reflect real altruism given that it entailed a real cost for the participants who exerted it^13,62,63^.

This result suggests that the motivations of men and women to engage in 3PP could be different, a difference very relevant to the role of 3PP in supporting extended prosociality. It would be interesting to study whether this result is related to the previously described tendency of women to behave in a care-oriented and context-dependent way, in contrast to the rigid norm-oriented, context-independent behavior that is more prevalent in men^64,65^. This differential way of evaluating third-parties could be driving the sex differences in 3PP described in the literature^15,29,30^, a feature we also observe in our sample even after controlling for sexually dimorphic characteristics. It is interesting to point out that 3PP, in addition to promoting an increase in general prosociality^66^, confers a gain in status and trustworthiness to the individual who performs it^8,10^. This gain might be more appealing to men according to the stronger association they face between reproductive success and status^67^. In contrast, second-party punishment does not award any of these advantages^66,68^. Further analyses on the motivation to engage in 3PP in men and women controlling for the tendency to display care-oriented, normative, and status-seeking behaviors could help to clarify the sexual differences in the association between 3PP and cooperation and, consequently, on cooperative behavior within groups^32^.

In fact, the existence of sex differences in the motivations to engage in 3PP is also supported by the relationships between sexually dimorphic features and 3PP we observe. In line with *Hypothesis 3*, we find an inverse association between facial dimorphism and 3PP in men (risk ratio: 0.76), which remains robust after controlling for related variables (PDGb, SPA). Let us highlight that the effect size of facial dimorphism on 3PP is similar to that of SPA, a self-evaluated variable. Higher facial masculinity is often related to lower prosociality in situations lacking social context^26,40–42^; the same holds for other features related to pubertal testosterone^60,69^. It thus makes sense that we observe higher facial dimorphism to be negatively related to 3PP in men since 3PP is considered to be a prosocial behavior. It is not infrequent to observe individuals who display features associated with a good physical condition to be less prosocial^22,24,25,39^. This is often explained by their alleged greater ability to obtain resources by themselves^17,70^.

The effect of SPA we describe supports the view of 3PP as a prosocial behavior: men who deem themselves as more attractive are more likely to engage in 3PP (risk ratio: 1.26). This agrees with previous studies showing that men who consider themselves attractive are more prosocial^38^. We also observe this relationship in our PDG: men with higher SPA are indeed more likely to cooperate (d = 0.186; Table 1). The effect of SPA in men’s behavior is consistent with the so-called “attractiveness halo effect”^71^, which states that more attractive people are treated better by others but are also expected to be more prosocial^72^.

In women, we expected no relationship between the degree of facial dimorphism and 3PP. This was based on the lack of a clear link between women’s facial dimorphism and prosocial/antisocial behavior^25,26,42^. The model in Table 2 shows a very weak relationship between facial dimorphism and 3PP in women (risk ratio: 1.06). In fact, this relationship is not significant at any standard confidence level before controlling for other variables (Table 1). SPA shows a negative effect on the likelihood to engage in 3PP when facial dimorphism and cooperation in the PDG are controlled for (risk ratio: 0.85). The standing literature offers conflicting results on the association between attractiveness and prosocial behavior in women; some studies show a negative association^25,38,60^, whereas others show no relationship at all^61,73^. Our study confirms these mixed results. We find no significant differences in SPA between women who punished and those who did not, but SPA shows a negative effect on 3PP in our final model. This difference is due to the fact that we control for PDG behavior: defection in the PDG is positively associated with 3PP (Table 2), and defecting women tend to see themselves as more attractive (d = 0.203; Table 1). Hence, once we control for behavior in the PDG, a negative relationship between SPA and 3PP emerges. It would be interesting to carry out further experiments on this complex relationship between SPA, prosociality and 3PP in women, with SPA being a variable of interest rather than just a control.

As mentioned, the sex differences we observe in the relationship between cooperation and 3PP and between facial dimorphism and 3PP suggest that men and women may have different underlying proximal or evolutionary motivations to engage in altruistic punishment. These differences could be worth exploring in the future; for instance, it would be interesting to understand better the differential impact of 3PP on fitness by sex or the influence of empathic mechanisms on this behavior. In any case, the lack of a common relationship between 3PP and cooperation across sexes indicates that 3PP might not be a mechanism enforcing cooperation within a community, but a form of exploitation. Further research must investigate how altruistic 3PP, a behavior exerted mostly and more intensively by half of the population, could support extended cooperation^5,34^ as some models suggest^74,75^ under different contexts^76,77^ and under different forms of punishment^78,79^. In this sense, let us reiterate that our results have been obtained studying exclusively a university population within a Western culture. It would be important to confirm them in a wider range of ages, ethnicities and socio-cultural strata. It is also worth investigating to what extent non-kin prosociality is motivated by social norms or by affective reactions, and whether this relationship differs between sexes^64,65^. It would also be interesting to delve deeper into the relationship between 3PP and other prosocial behaviors in order to establish whether 3PP exerted against an individual who harms others can be indeed considered prosocial^14^.

Some of the variables we expected to correlate with 3PP were not significant. We found no support in favour of *Hypothesis 2*: 2D:4D did not show any influence on behavior in either sex. Previous literature on 2D:4D and prosociality indicates that their association is non-linear^21,22^, and that 2D:4D mediates the effect of other variables depending on the context^27,53,54,80,81^ Future experiments varying the context in which decisions are made might uncover an effect of 2D:4D on 3PP. We do not find any evidence either of a direct effect of FA on 3PP (*Hypothesis 4*), another variable previously related to prosociality^25,82^. This lack of a direct association indicates, once again, that the relationship between prosociality and 3PP is more complex than usually conjectured. Nonetheless, we observe a weak association between cooperation and FA in men (d = 0.067; Table 1) and between cooperation and punishment (Table 2). These results are consistent with the conjecture that altruistic punishment is a prosocial behavior in men but not in women.

We would like to emphasize at this point that although we chose our variables of interest considering that prosociality is stable over time and that individuals tend to behave prosocially in different contexts, this does not mean that prosociality must be considered as a trait. Stability across time and context could also result from prosociality being a group of correlated mechanisms leading to behaviors aimed at improving social welfare. In fact, our results suggest that prosociality is not a single trait, given that the set of variables related to the tendency to engage in altruistic 3PP and of those previously described to be related to prosociality do not overlap.

Before concluding, let us highlight a byproduct of our study. We carried our analysis in a very large sample, allowing us to test further the relationship between cooperation and some individual features. Our analysis confirm some of the results observed in moderate sample sizes, such as the relationship between FA and cooperation in men^22,83^, and the positive (negative) correlation between SPA and cooperation in men (women)^25,38^. We found no differences in the other variables considered (facial dimorphism, 2D:4D) between cooperators and non-cooperators in either men or women (Table 1).

## Conclusions

Our findings provide new insights on the relationship between cooperation and altruistic punishment. There exist sex differences in the tendency to engage in 3PP, with cooperating men being more likely to punish. This contrasts with what we observe in women. We also explore how pubertal levels of sexual hormones influence altruistic punishment: as expected, there is an inverse association between facial dimorphism and 3PP in men, whereas the association is much weaker or non-existent in women. These findings suggest that the motivations for performing 3PP are different in men and women. We hope to have broadened the debate on the underlying causes of prosociality and their nuances.

## Methods

### Participants

839 students from Universidad Autónoma de Madrid (Spain) participated in the experiment (511 women and 328 men) once only. Mean age (±SD) of women was 21.09 ± 2.57, and mean age of men was 21.82 ± 2.32. All participants included in this study classified themselves as Caucasian. Data from participants belonging to other ethnicities, marginally represented in this population, were collected but discarded because they introduced facial shape variations stronger than sexual dimorphism in the analysis.

### Experimental session

Each session was carried out with 8–20 same-sex participants. Before the experiment took place, subjects were instructed about the procedures. They filled in a questionnaire eliciting their ethnicity and self-perceived attractiveness (SPA), measured in a slider scale. The experimental procedures received approval from the Universidad Autónoma de Madrid (UAM) Ethics Committee (approval number: CEI 73–1319). All the methods were performed in accordance with the relevant guidelines and regulations of the institution, and informed consent of all the participants was obtained.

The experiment was programmed using the z-Tree 3.2.10 software for economic experiments^84^. In the first stage, participants played a prisoner’s dilemma game (PDG), a strategic game widely used to measure cooperation^12,19,22,24^. They had to decide whether to cooperate or not with an anonymous counterpart. This counterpart was an anonymous participant in a previous session. We informed participants that their decisions would be employed in future sessions as anonymous potential counterparts. We also asked our participants about the behavior they expected from their counterpart. Participants were not informed about the result of this interaction. In the second stage, participants played as observers. They were presented a PDG between two other individuals. Participants were asked what they would do if one of these individuals chose not to cooperate whilst declaring that he/she expected his/her counterpart to cooperate. The participant had to decide whether he/she would pay a cost to reduce the earnings of this defector but without knowing the actual outcome of the PDG they were observing. The individuals playing the PDG observed by our participants were subjects of future experimental sessions, so their punishment decisions had monetary consequences. We implemented the decisions of observers in a separate series of sessions whose data is not included in the present analysis.

Participants were paid according to their choices during the entire session, which included other experiments not analysed in the present study. In the PDG stage, payoffs were as follows: if they both cooperated, participants got 90 points; if none of them cooperated, they both got 30 points; if one of them cooperated and the other did not, the cooperator got 10 points, and the defector 160 points. In the 3PP stage, participants were assigned 80 points and were asked whether they would pay half of these points (40 points) to decrease by 120 points the total earnings of the uncooperative participant they were observing. Conversion from points to euros was 1€/100 points. The size of stakes was thus relatively small, a feature which could affect strategic behavior^85^. We nonetheless employed these values as they had already been successfully implemented in similar studies^22,25^. Let us emphasize that our design allows participants to engage in truly altruistic punishment^13,62,63^. There was only one punishing stage, so there was no strategic motivation to exert 3PP; that is, there were no additional future interactions that participants could take into account when making their decisions. In addition, participants could not use 3PP to build up reputation because their choice was one-shot and was not made public. Their motivation could not be revenge either, since participants could not punish individuals they had interacted with at any moment. Punishments could not be affected either by distributional concerns such as spite or inequality aversion, as participants were not informed of the outcome of the PDG they were observing. Finally, let us reiterate that punishment decisions were costly to our participants and had an actual effect on other participants; they were not hypothetical and had real consequences.

### Measurements

We obtained hand-scans from each participant with a CanoScan LiDE 200 scanner as well as two frontal facial photographs in standardized conditions. Lengths of the second and fourth digits were measured from the hand scans as it is customary in the literature^21,86,87^. We computed their ratios, averaged the two resulting numbers, and included them in the analysis. In order to compute the facial FA and facial dimorphism measures, two independent observers placed 39 predefined landmarks in the two photographs of each participant with the TPS 2.16 software (by F.J. Rohlf; obtained from http://life.bio.sunysb.edu/ee/rohlf/software.html). Morphometric analyses were carried out with MorphoJ 1.04a (by C.P. Klingenberg; obtained from http://www.flywings.org.uk/morphoj_page.htm). The Mahalanobis FA score was computed, using the methods described in the literature^22^. Dimorphism scores were obtained by a discriminant score as described in previous studies^37^; women’s scores were converted to positive values to obtain an absolute value of facial dimorphism. Higher scores indicate higher facial shape differences from the other sex. Descriptive statistics for men and women are shown in Table 3.

**Table 3:** Descriptive statistics by sex. The variables included are Dimorphism (facial dimorphism), FA (Fluctuating asymmetry), 2D:4D (average of left and right second to fourth digit ratio), SPA (self-perceived attractiveness). 95% CI: 95% confidence interval for each mean.

	Sex	N	Mean ± 95% CI	t	Df	p	d
Dimorphism	Women	511	4.59 ± 0.27	−0.63	837	0.529	−0.045
	Men	328	4.72 ± 0.34				
FA	Women	511	3.04 ± 0.05	5.63	837	< 0.001	0.398
	Men	328	2.84 ± 0.05				
2D:4D	Women	511	0.978 ± 0.003	5.90	837	< 0.001	0.418
	Men	328	0.965 ± 0.003				
SPA	Women	511	0.608 ± 0.013	−0.10	837	0.918	−0.007
	Men	328	0.609 ± 0.021				

### Statistical analyses

Statistical analyses were carried out with SPSS 15.0. We checked the normality of all variables and of the residuals in all models.

We analysed the relationship between biological variables and behavior by means of t-tests. The effect of squared 2D:4D was tested simultaneously with that of 2D:4D^22^ in a logistic regression, but the effect was not significant (Supplementary Table S6).

Models were built following standard recommendations^88,89^, namely testing each variable independently and introducing all the significant ones and those with a strong theoretical support in one model for men and women separately. We included the choice and the expected behavior of the counterpart in the first stage PDG, considering the behavior displayed as a measure of cooperativeness. Next, we tested those variables in a single model including their interaction with sex, searching for the simpler significant model. Finally, we checked the interactions between the remaining variables.

As it is recommended for logistic models^90^, we computed effect sizes for all variables in the form of risk ratios, i.e. the ratio between the probability of the event before and after applying a change in the selected variable. For continuous variables, the change consists of a one standard deviation increase from the mean of the variable. For dichotomous variables, the change is just from 0 to 1. A comparison between the effect size after and before their inclusion in the model are showed in Supplementary Fig. S1.

## Supplementary information

Supplementary file

Supplementary Dataset 1

## Data Availability

Data from this study is available as Supplementary Material.
