# Supplementary material for: Sex Affects the Relationship Between Third Party Punishment and Cooperation
Source: Sci Rep. 2019 Mar 12;9:4288. doi: 10.1038/s41598-019-40909-8 (PMC6414674; doi:10.1038/s41598-019-40909-8)

**SUPPLEMENTARY MATERIAL FROM “SEX AFFECTS THE RELATIONSHIP BETWEEN THIRD PARTY PUNISHMENT AND COOPERATION”**

Claudia Rodríguez-Ruiz, José Antonio Muñoz-Reyes, Marta Iglesias-Julios, Santiago Sanchez-Pages, Enrique Turiegano

SUPPLEMENTARY TABLE 1: Proportion of males and females by behavior in the PDG stage (cooperation and expectation of cooperation) and in the 3PP stage.

|  |  | **Proportions per category** | | **Proportions in full sample** | |
| --- | --- | --- | --- | --- | --- |
|  |  | **Women** | **Men** | **Women** | **Men** |
| **Cooperate** | **Do not punish** | 81.98% | 71.08% | 55.19% | 44.21% |
|  | **Punish** | 18.02% | 28.92% | 12.13% | 17.99% |
| **Defect** | **Do not punish** | 74.85% | 78.23% | 24.46% | 29.57% |
|  | **Punish** | 21.15% | 21.77% | 8.22% | 8.23% |
|  |  | Χ^2^_1_=3.522; p=0.061 | Χ^2^_1_=2.037; p=0.154 | Χ^2^_3_=11.452; p=0.010 | |
| **Expect cooperation** | **Do not punish** | 81.71% | 75.23% | 55.97% | 49.08% |
|  | **Punish** | 18.28% | 24.77% | 12.52% | 16.16% |
| **Expect defection** | **Do not punish** | 75.16% | 71.05% | 23.68% | 24.70% |
|  | **Punish** | 24.84% | 28.95% | 7.83% | 10.06% |
|  |  | Χ^2^_1_=2.927; p=0.087 | Χ^2^_1_=0.672; p=0.412 | Χ^2^_3_=4.899; p=0.179 | |

**Procedure followed to build the final model**

We first built a logistic model for men with the decision to engage in 3PP as the dependent variable (supplementary table 2), introducing facial dimorphism and self-perceived attractiveness as independent variables. Both variables emerged as significant but not their interaction. We then controlled for the participants’ behaviour in the PDG. The choice to cooperate (PDGb) was significant, but not the belief about the decision of the counterpart (PDGeb) nor their interaction. For women, we built a model including the same variables which were significant for men. In this model, no physiology-related variable affected 3PP (supplementary table 2). Only the behaviour in the PDG was significant, showing the opposite effect than in men, that is, indicating that non-cooperative women were more prone to punish in the 3PP stage.

We then pooled all observations and ran three separate logistic regression models (supplementary table 3) including the sex dummy and each of the three variables previously observed to be significant (PDGb, sexual dimorphism and SPA) together with their interaction with sex. All three variables and their interaction with the sex dummy were significant. To analyse them simultaneously, we ran a fourth model including sex, PDGb, facial dimorphism and SPA, and their interactions with the sex dummy. All the variables showed a significant effect on punishing behaviour mediated by sex. All additional interactions were not significant (supplementary table 4) and thus were excluded from the final model.

SUPPLEMENTARY TABLE 2: Logistic models for punishment by sex. The variables included in the final model are Dimorphism (facial dimorphism), SPA (self-perceived attractiveness), and the categorical variables PDGb and PDGeb (behavior and expected behavior in the PDG). The reference values for the categorical variables are male for sex and cooperate for PDGb and PDGeb. ^+^ calculated with respect to the first model

| **Variables in the model** | **MODEL** | | | | **VARIABLE** | | | | |  |
| --- | --- | --- | --- | --- | --- | --- | --- | --- | --- | --- |
|  | -2LL | Likelihood Ratio Test | df | P | variables | coef | Wald | df | P |  |
| *Dimorphism, SPA* | 363.30 | 14.12 | 2 | 0.001 | Constant | -1.797 | 14.23 | 1 | <0.001 | **MEN** |
|  |  |  |  |  | Dimorphism | -0.110 | 6.69 | 1 | 0.010 |  |
|  |  |  |  |  | SPA | 0.288 | 8.31 | 1 | 0.004 |  |
| *Dimorphism, SPA, PDG b, PDG eb,  PDG b x PDG eb* | 357.23 | 6.07^+^ | 3^+^ | 0.108 | Constant | -1.680 | 11.22 | 1 | 0.001 |  |
|  |  |  |  |  | Dimorphism | -0.105 | 5.96 | 1 | 0.015 |  |
|  |  |  |  |  | SPA | 0.275 | 7.39 | 1 | 0.007 |  |
|  |  |  |  |  | *PDG b* | -1.160 | 4.92 | 1 | 0.037 |  |
|  |  |  |  |  | *PDG eb* | 0.147 | 0.12 | 1 | 0.719 |  |
|  |  |  |  |  | *PDG b x PDG eb* | 0.944 | 1.92 | 1 | 0.165 |  |
| *Dimorphism, SPA, PDG b, PDG eb,  PDG b x PDG eb* | 509.13 | 7.23 | 5 | 0.204 | Constant | -0.961 | 3.83 | 1 | 0.050 | **WOMEN** |
|  |  |  |  |  | Dimorphism | 0.030 | 0.69 | 1 | 0.406 |  |
|  |  |  |  |  | SPA | -0.177 | 2.59 | 1 | 0.108 |  |
|  |  |  |  |  | *PDG b* | -0.303 | 0.55 | 1 | 0.457 |  |
|  |  |  |  |  | *PDG eb* | -0.152 | 0.03 | 1 | 0.859 |  |
|  |  |  |  |  | *PDG b x PDG eb* | -0.057 | 0.01 | 1 | 0.916 |  |
| *Dimorphism, SPA, PDG b* | 509.91 | -0.78 | 2^+^ | 0.676 | Constant | -0.920 | 3.60 | 1 | 0.058 |  |
|  |  |  |  |  | Dimorphism | 0.030 | 0.68 | 1 | 0.410 |  |
|  |  |  |  |  | SPA | -0.177 | 2.57 | 1 | 0.109 |  |
|  |  |  |  |  | *PDG b* | 0.457 | 3.98 | 1 | 0.046 |  |
| *PDG b* | 512.93 | -3.80 | 4^+^ | 0.434 | Constant | -1.515 | 116.62 | 1 | <0.001 |  |
|  |  |  |  |  | *PDG b* | 0.424 | 3.49 | 1 | 0.062 |  |

SUPPLEMENTARY TABLE 3. Pooled logistic models for 3PP. The variables initially included are Dimorphism (facial dimorphism), SPA (self-perceived attractiveness), and the categorical variables sex and PDGb (behavior in the PDG). The reference values for the categorical variables are male for sex and cooperate for PDGb. ^+^ Calculated with respect to the model without interactions.

| **Variables in the model** | **MODEL** | | | | **VARIABLE** | | | | |
| --- | --- | --- | --- | --- | --- | --- | --- | --- | --- |
|  | -2LL | Likelihood Ratio Test ^+^ | Df ^+^ | p | Variables | Coef | Wald | Df | p |
| *Sex, PDG b,  Sex x PDG b* | 888.280 | 5.32 | 1 | 0.021 | Constant | -0.899 | 33.91 | 1 | <0.001 |
|  |  |  |  |  | Sex | -0.616 | 8.71 | 1 | 0.003 |
|  |  |  |  |  | PDG b | -0.380 | 2.03 | 1 | 0.155 |
|  |  |  |  |  | Sex x PDG b | 0.804 | 5.27 | 1 | 0.022 |
| *Sex, Dimorphism,  Sex x Dimorphism* | 887.842 | 4.94 | 1 | 0.026 | Constant | -0.601 | 7.49 | 1 | 0.006 |
|  |  |  |  |  | Sex | -0.884 | 8.72 | 1 | 0.003 |
|  |  |  |  |  | Dimorphism | -0.096 | 5.32 | 1 | 0.021 |
|  |  |  |  |  | Sex x Dimorphism | 0.122 | 4.89 | 1 | 0.027 |
| *Sex, SPA,  Sex x SPA* | 888.618 | 3.93 | 1 | 0.048 | Constant | -2.157 | 22.70 | 1 | <0.001 |
|  |  |  |  |  | Sex | 1.413 | 4.70 | 1 | 0.030 |
|  |  |  |  |  | SPA | 0.257 | 6.70 | 1 | 0.008 |
|  |  |  |  |  | Sex x SPA | -0.404 | 7.64 | 1 | 0.006 |
| *Sex, PDG b, Dimorphism, SPA, Sex x PDG b, Sex x Dimorphism, Sex x SPA* | 871.827 | 11.89 | 3^+^ | 0.007 | Constant | -1.662 | 11.33 | 1 | 0.001 |
|  |  |  |  |  | Sex | 0.742 | 1.15 | 1 | 0.284 |
|  |  |  |  |  | PDG b | -0.312 | 1.31 | 1 | 0.252 |
|  |  |  |  |  | Dimorphism | -0.109 | 6.52 | 1 | 0.011 |
|  |  |  |  |  | SPA | 1.969 | 7.80 | 1 | 0.005 |
|  |  |  |  |  | Sex x PDG b | 0.769 | 4.67 | 1 | 0.031 |
|  |  |  |  |  | Sex x Dimorphism | 0.139 | 6.17 | 1 | 0.013 |
|  |  |  |  |  | Sex x SPA | -3.208 | 9.40 | 1 | 0.002 |

SUPPLEMENTARY TABLE 4. Additional interactions for the pooled logistic models for 3PP. The variables initially included were Dimorphism (facial dimorphism), SPA (self-perceived attractiveness), the categorical variables PDGb (behavior in the PDG) and sex, and the interaction of the first three variables with sex. The reference values for the categorical variables are male for sex and cooperate for PDGb. ^+^ Calculated with respect to the final model.

| **Variables in the model** | **MODEL** | | | | **VARIABLE** | | | | |
| --- | --- | --- | --- | --- | --- | --- | --- | --- | --- |
|  | -2LL | Likelihood Ratio Test | Df | p | Variables | Coef | Wald | Df | p |
| *Sex, PDG b, Dimorphism, SPA, Sex x PDG b, Sex x Dimorphism, Sex x SPA,*  *Dimorphism x SPA* | 871.684 | 0.14^+^ | 1^+^ | 0.705 | Constant | -1.845 | 7.04 | 1 | 0.008 |
|  |  |  |  |  | Sex | 0.766 | 1.20 | 1 | 0.273 |
|  |  |  |  |  | PDG b | -0.305 | 1.24 | 1 | 0.265 |
|  |  |  |  |  | Dimorphism | -0.069 | 0.37 | 1 | 0.544 |
|  |  |  |  |  | SPA | 2.262 | 4.63 | 1 | 0.031 |
|  |  |  |  |  | Sex x PDG b | 0.768 | 4.65 | 1 | 0.031 |
|  |  |  |  |  | Sex x Dimorphism | 0.136 | 5.72 | 1 | 0.017 |
|  |  |  |  |  | Sex x SPA | -3.224 | 9.48 | 1 | 0.002 |
|  |  |  |  |  | Dimorphism x SPA | -0.063 | 0.14 | 1 | 0.706 |
| *Sex, PDG b, Dimorphism, SPA, Sex x PDG b, Sex x Dimorphism, Sex x SPA,*  *PDGb x Dimorphism* | 870.919 | 0.91^+^ | 1^+^ | 0.341 | Constant | -1.556 | 9.48 | 1 | 0.002 |
|  |  |  |  |  | Sex | 0.771 | 1.24 | 1 | 0.265 |
|  |  |  |  |  | PDG b | -0.546 | 2.20 | 1 | 0.138 |
|  |  |  |  |  | Dimorphism | -0.129 | 7.27 | 1 | 0.007 |
|  |  |  |  |  | SPA | 1.937 | 7.56 | 1 | 0.006 |
|  |  |  |  |  | Sex x PDG b | 0.750 | 4.41 | 1 | 0.036 |
|  |  |  |  |  | Sex x Dimorphism | 0.135 | 5.76 | 1 | 0.016 |
|  |  |  |  |  | Sex x SPA | -3.219 | 9.46 | 1 | 0.002 |
|  |  |  |  |  | PDGb x Dimorphism | 0.053 | 0.91 | 1 | 0.341 |
| *Sex, PDG b, Dimorphism, SPA, Sex x PDG b, Sex x Dimorphism, Sex x SPA,*  *PDGb x SPA* | 871.049 | 0.78^+^ | 1^+^ | 0.378 | Constant | -1.924 | 10.85 | 1 | 0.001 |
|  |  |  |  |  | Sex | 0.761 | 1.18 | 1 | 0.277 |
|  |  |  |  |  | PDG b | 0.273 | 0.15 | 1 | 0.703 |
|  |  |  |  |  | Dimorphism | -0.108 | 6.33 | 1 | 0.012 |
|  |  |  |  |  | SPA | 2.361 | 7.87 | 1 | 0.005 |
|  |  |  |  |  | Sex x PDG b | 0.739 | 4.27 | 1 | 0.039 |
|  |  |  |  |  | Sex x Dimorphism | 0.139 | 6.20 | 1 | 0.013 |
|  |  |  |  |  | Sex x SPA | -3.199 | 9.29 | 1 | 0.002 |
|  |  |  |  |  | PDGb x SPA | -0.924 | 0.78 | 1 | 0.378 |

SUPPLEMENTARY TABLE 5: Descriptive statistics by participant’s expected behavior in the PDG stage. The variables are Dimorphism (facial dimorphism), FA (Fluctuating asymmetry), 2D:4D (average of left and right second to fourth digit ratio), SPA (self-perceived attractiveness). Data are presented as mean ± SD (95% confidence interval).

|  | **WOMEN** | | **MEN** | |
| --- | --- | --- | --- | --- |
|  | **Exp. defect. (N=161)** | **Exp. coop. (N=350)** | **Exp. defect. (N=114)** | **Exp. coop. (N=214)** |
| **Dimorphism** | 4.61±3.31  (4.17-5.16) | 4.58±2.92  (4.22-4.84) | 4.71±3.25  (4.12-5.30) | 4.73±2.98  (4.34-5.16) |
|  | U=28905, p=0.638, d=0.013 | | U=19010, p=0.109, d=-0.079 | |
| **FA** | 3.06±0.53  (2.96-3.10) | 3.04±0.55  (2.92-3.03) | 2.87±0.48  (2.76-2.91) | 2.83±0.42  (2.75-2.86) |
|  | U=29692, p=0.328, d=0.039 | | U=20905, p=0.848, d=0.001 | |
| **2D:4D** | 0.975±0.031  (0.969-0.979) | 0.979±0.032  (0.976-0.982) | 0.967±0.030  (0.961-0.972) | 0.964±0.029  (0.960-0.968) |
|  | U=25710, p=0.112, d=-0.144 | | U=19158, p=0.136, d=-0.182 | |
| **SPA** | 0.621±0.160  (0.581-0.646) | 0.602±0.135  (0.577-0.630) | 0.602±0.207  (0.568-0.642) | 0.613±0.183  (0.580-0.644) |
|  | U=31099, p=0.059, d=0.134 | | U=23260, p=0.119, d=0.148 | |

SUPPLEMENTARY TABLE 6: Effect of 2D:4D and its square on 3PP by sex.

|  | **MODEL** | | | | **VARIABLE** | | | | |
| --- | --- | --- | --- | --- | --- | --- | --- | --- | --- |
|  | -2LL | Likelihood Ratio Test | Df | p | Variables | Coef | Wald | Df | p |
| *WOMEN* | 513.385 | 2.98 | 2 | 0.226 | Constant | 28,868 | 0,16 | 1 | 0,687 |
|  |  |  |  |  | 2D:4D | -67,174 | 0,21 | 1 | 0,644 |
|  |  |  |  |  | 2D:4D^2^ | 37,028 | 0,25 | 1 | 0,616 |
| *MEN* | 337.423 | 0.43 | 2 | 0.808 | Constant | 59,843 | 0,41 | 1 | 0,523 |
|  |  |  |  |  | 2D:4D | -126.546 | 0,43 | 1 | 0,514 |
|  |  |  |  |  | 2D:4D^2^ | 65,701 | 0,43 | 1 | 0,512 |

SUPPLEMENTARY FIGURE 1:

Comparison of the effect sizes on 3PP of the variables considered, evaluated alone and in the final model. The grey 45° line indicates identical effect sizes. Odd ratios (OR) for each variable were calculated employing the final logistic model in table 2. OR for the variables evaluated alone are approximated using Cohen’s d (from table 1) or phi following the procedures described in Sanchez-Meca et al. (2003), which assume that each of the two continuous populations follows a logistic distribution with equal variances.


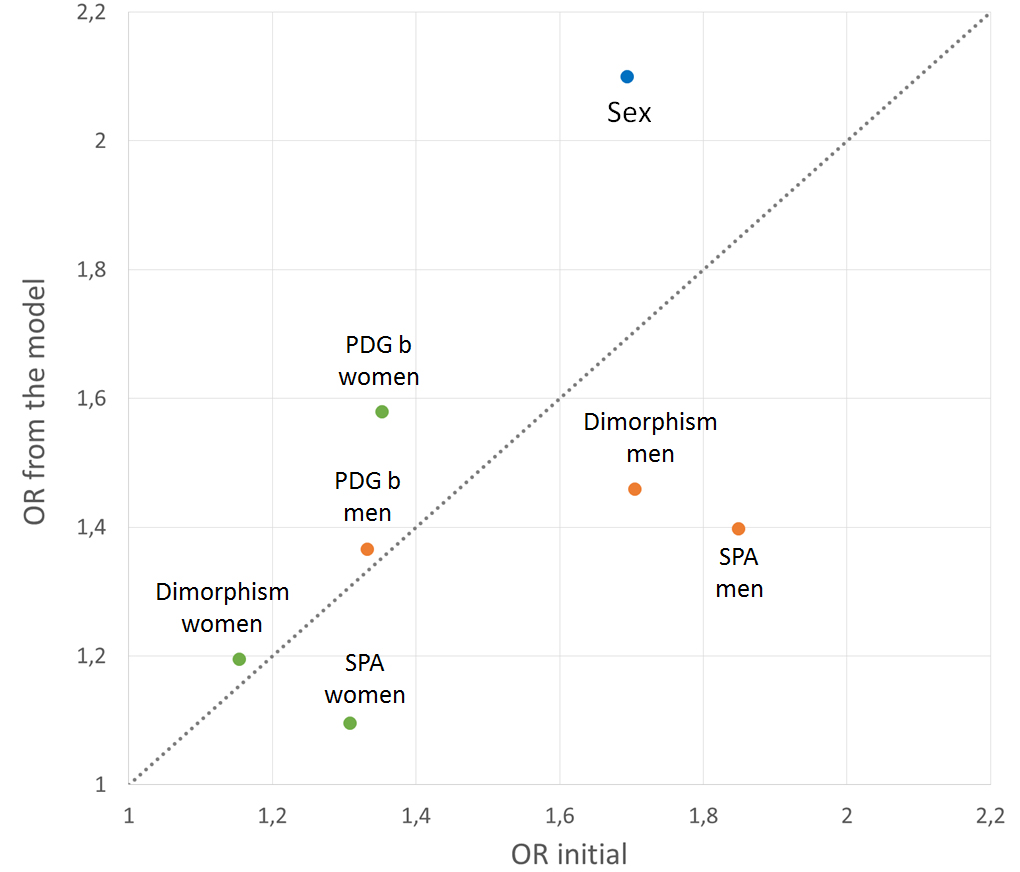

Supplement: Supplementary file 1 — Supplementary file [file 41598_2019_40909_MOESM1_ESM.docx]
